# Environmental Temperatures Affect the Gastrointestinal Microbes of the Chinese Giant Salamander

**DOI:** 10.3389/fmicb.2021.543767

**Published:** 2021-03-19

**Authors:** Lifeng Zhu, Wei Zhu, Tian Zhao, Hua Chen, Chunlin Zhao, Liangliang Xu, Qing Chang, Jianping Jiang

**Affiliations:** ^1^College of Life Science, Nanjing Normal University, Nanjing, China; ^2^Chengdu Institute of Biology, Chinese Academy of Sciences, Chengdu, China; ^3^Mingke Biotechnology Co., Ltd., Hangzhou, China

**Keywords:** global warming, gastrointestinal microbiome, longitudinal analysis, alpha and beta diversity, body growth, environmental temperatures

## Abstract

An increasing number of studies have shown that warming also influences the animal gut microbiome (altering the community structure and decreasing its diversity), which might further impact host fitness. Here, based on an analysis of the stomach and gut (the entire intestine: from the anterior intestine to the cloaca) microbiome in laboratory larva of giant salamanders (*Andrias davidianus*) under different living water temperatures (5, 15, and 25°C) at two sample time points (80 and 330 days after the acclimation), we investigated the potential effect of temperature on the gastrointestinal microbiome community. We found the significant Interaction between sampling time and temperature, or type (stomach and gut) on Shannon index in the gastrointestinal microbiome of the giant salamanders. We also found the significant difference in Shannon index among temperature groups within the same sample type (stomach or gut) at each sample time. 10% of variation in microbiome community could be explained by temperature alone in the total samples. Both the stomach and gut microbiomes displayed the highest similarity in the microbiome community (significantly lowest pairwise unweighted Unifrac distance) in the 25-degree group between the two sampling times compared to those in the 5-degree and 15-degree groups. Moreover, the salamanders in the 25°C treatment showed the highest food intake and body mess compared to that of other temperature treatments. A significant increase in the abundance of Firmicutes in the gastrointestinal microbiome on day 330 with increasing temperatures might be caused by increased host metabolism and food consumption. Therefore, we speculate that the high environmental temperature might indirectly affect both alpha and beta diversity of the gastrointestinal microbiome.

## Introduction

The holobiont (the host plus all of its microbes) can function as a distinct biological system and plays an important role in metabolism, immunity, and development (Margulis and Fester, 1991; Rosenberg and Zilber-Rosenberg, 2018). Climate warming has led to a profound threat to amphibian populations (Beebee and Griffiths, 2005; Pounds et al., 2006; Alford et al., 2007). Three interesting studies have found an effect of environmental temperature on the gut microbial communities of ectothermic amphibians and reptiles. For example, one study revealed that the gut microbial communities of laboratory raised tadpoles of the northern leopard frog (*Lithobates pipiens*) in warm (28°C) temperature treatments exhibited a higher relative abundance of the phylum Planctomycetes and Actinobacteria and lowered relative abundance of Firmicutes and Proteobacteria than in the cool treatment (18°C) (Kohl and Yahn, 2016). Changes in environmental temperature, and those mediated through changes in host physiology, may result in significant changes in the gut microbial community (Kohl and Yahn, 2016). Furthermore, the gut microbiome in red-backed salamanders housed at three experimental temperatures (10, 15, and 20°C) showed a decrease in microbial diversity among these treatments (Fontaine et al., 2018). A third study found that +2–3°C increase in temperature resulted in a 34% loss of populations’ microbiota diversity (number of OTUs) in the wild ectothermic common lizard *Zootoca vivipara* (Bestion et al., 2017). Differing environmental temperature have also been correlated with changes in the gut microbiota of other animals, such as the spring field cricket *Gryllus veletis* (Ferguson et al., 2018), and the Brandt’s voles (*Lasiopodomys brandtii*) (Zhang et al., 2018). Therefore, understanding the threat to animal biodiversity posed by global warming will require not only studying the animal but also their gastrointestinal microbiome.

The gastric microbiome also plays an important role in host nutrition and health (Hungate, 1966; King et al., 2012; Hollister et al., 2014). Studies on gastrointestinal microbiome will provide more information on the potential effect by environmental factors (e.g., temperature) compared to those using a single type of microbes. The stomach is the link between the esophagus and the beginning of the small intestine, and its role is to digest food (partially) by mechanical and chemical digestion (e.g., gastric acids, lipase, and digestive enzymes) and send it to the small intestine. The function of the small intestine is to absorb nutrients through its inner surface and send them into the bloodstream (Sandblom, 1970). The differences in conditions, along with the food or other ingested substrates between the stomach and intestine, affect the composition and function of the microbes (Hillman et al., 2017; Spohn and Young, 2018). However, will the environmental temperature impact the stomach and gut microbiomes on the same time, and are there some common patterns in the changes between the stomach and gut microbiome? Then, what will happen to the microbiome community in the gastric and gut over time, along with the different environmental temperatures, and can these changes be maintained for long periods?

The Chinese giant salamander (*Andrias davidianus*), the largest extent amphibian, is a flagship species for amphibian biodiversity conservation. These salamanders typically experience temperatures of 8–25°C, with 15–21°C as optimal for somatic growth (Hill et al., 1975; Hutchison and Hill, 1976). And temperature higher than 28°C would threaten the survive of these animals (Mu et al., 2011). The captive individuals have prominent cold preference, with behavioral preference temperature lower than the reported optimal growth temperature in aquaculture (15–21°C), and warm acclimation can’t improve their preferred temperature (our unpublished data). These results suggest that the Chinese giant salamander may be sensitive to global warming. Our previous study found age-related changes in the gastrointestinal microbiota of a captive Chinese giant salamander population (*A. davidianus*), with the stomach and gut showing different microbial communities (Zhang et al., 2018). The stomach in 3 years old salamanders harbors a relatively high relative abundance of Proteobacteria (e.g., genus *Aeromonas*) and Tenericutes (e.g., *Mycoplasma*), while the gut has a high relative abundance of Fusobacteria and Firmicutes (Zhang et al., 2019). In the present study, we investigate the changes in the gastrointestinal microbiome in a laboratory Chinese giant salamander population under the same diet and across different environmental temperatures (5, 15, and 25°C) across two sampling times (80 and 330 days after the acclimation). Moreover, to explore the putative host-microbe interaction at different temperatures, we also measured their body growth during the experiment.

## Results

We gained the sequencing data from 47 samples belonging to 63 individuals (the range of sequencing reads: from 30,622 to 57,960) in this study. On day 80, each temperature treatment group had three pooled samples for either stomach or gut. On day 330, we successfully gained the sequence from 13 stomach samples (5°C: one sample; 15°C: six samples; 25°C: six samples) samples and 16 gut samples (5°C: six samples; 15°C: four samples; 25°C: six samples). The total number of OTUs (Operational taxonomic units) was 1,676. After rarefaction (28,202 sequences per sample), we finally gained 1,517 OTUs.

### The Differences in the Gastrointestinal Microbial Groups Under Different Temperatures

**FIGURE 1:**
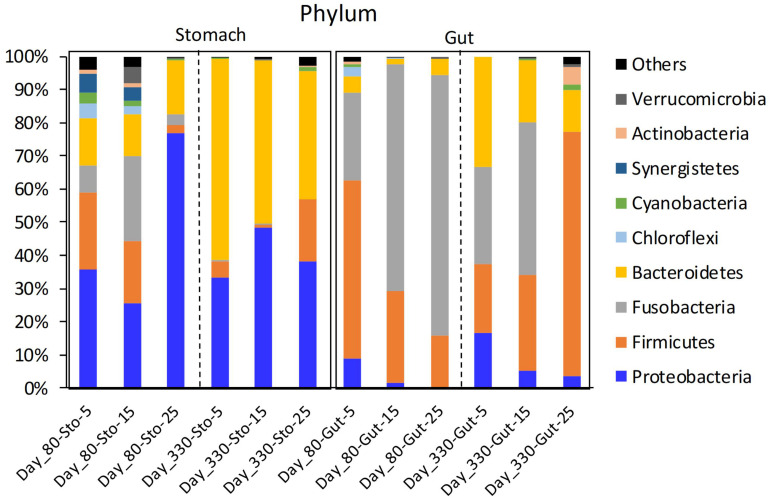
The mean relative abundance of the phylum in giant salamander stomach and gut microbiome under different temperatures. Sto, stomach content sample; Day 80, 80 days after acclimation; Day 330, 330 days after acclimation; Others, the total of the low relative abundance phyla.

The stomach microbiome of the Chinese giant salamanders mainly included Proteobacteria and Bacteroidetes. The gut microbiome of the Chinese giant salamanders mainly included Firmicutes, Fusobacteria, and Bacteroidetes (Figure 1). In the stomach samples on day 80, the relative abundance of Cyanobacteria was the enriched in the 5-degree group, the relative abundance of Fusobacteria was the enriched in the 15-degree group, and the relative abundance of Armatimonadetes was enriched in the 25-degree group (Figure 1 and Supplementary Figure 1). In the gut samples on 80 days after the acclimation (Day 80), we found the relative abundance of many phyla decreased along with the increasing of the environmental temperatures. For example, the relative abundance of Cyanobacteria, Proteobacteria, Planctomycetes, Actinobacteria, and Chloroflexi, were enriched in the 5-degree group (Figure 1 and Supplementary Figure 2). In the stomach samples on 330 days after the acclimation (Day 330), the relative abundances of many phyla (including Firmicutes, Cyanobacteria, Planctomycetes, Actinobacteria, and Tenericutes) were enriched in the 25-degree group (Figure 1 and Supplementary Figure 3). Here, we didn’t include the 5-degree group due to the only one sample in this group. In the gut samples on Day 330, the relative abundance of Proteobacteria was the enriched in the 5-degree group, the abundances of Cyanobacteria and Fusobacteria were the enriched in the 5-degree group, and the relative abundances of four phyla (including Firmicutes, Verrucomicrobia, Actinobacteria, and Tenericutes) were enriched in the 25-degree group, (Figure 1 and Supplementary Figure 4). Moreover, based on the significant differences in the relative abundance of the microbiome among the groups using LEfSe analysis, we estimated the four kinds of trends in the microbiome (genus level) observed in the microbiome with changing temperatures (Supplementary Figure 5).

First, we estimated the decreasing pattern in the relative abundance of some genera with increasing temperature (Supplementary Figure 5A). Two common genera were found in the stomach and gut microbiomes: *Limnohabitans* (Stomach: from 13.661% to 0.745%; Gut: from 0.281% to 0.027%) and *Methyloparacoccus* (Stomach: from 1.125% to 0.074%; Gut: from 0.233% to 0.006%). In the gut samples on day 80, the relative abundance of *Eubacterium* sharply decreased. During the second sampling period, no common genera were found between the stomach and gut samples. Second, we observed the increasing trend in the relative abundance of some genera with increasing temperature (Supplementary Figure 5B). Only one common genus was found in the stomach and gut microbiome: *Paraclostridium* (Stomach: from 0.008 to 0.113%; Gut: from 0.038 to 0.641%). In the stomach samples on day 80, the relative abundance of *Aeromonas* sharply increased (from 0.015 to 37.690%). During the second sampling period, ten common genera were found between the stomach and gut samples, namely *Akkermansia*, *Alistipes*, *Clostridium sensu stricto 1*, *Dielma*, *Hydrogenoanaerobacterium*, *Intestinibacter*, *Intestinimonas*, *Oscillibacter*, *Peptococcus*, and *Ruminococcaceae UCG-014*. Third, we observed convex trends in the relative abundance of some genera with increasing temperature (Supplementary Figure 5C). No common genera were found between the stomach and gut samples. The relative abundance of *Flavobacterium* was the highest (5-degree: 1.550%; 15-degree: 37.516%; 25-degree: 1.471%) in the stomach samples at 15-degree. The relative abundance of *Cetobacterium* was highest (5-degree: 29.057%; 15-degree: 46.935%; 25-degree: 0.024%) in the gut samples at 15-degree. Finally, we found the concave trend in the relative abundance of some genera with increasing temperature (Supplementary Figure 5D). In the stomach samples on day 80, the relative abundance of *Aquabacterium* in the stomach samples had the lowest relative abundance at 15 (5: 0.307%; 15: 0.124%; 25: 7.823%) on day 80. One genus (*Caproiciproducens*) with a concave trend was found in the gut on day 80. During the second sampling period, only one common genus (*Parabacteroides*) was found between the stomach and gut samples.

### Alpha Diversity Differences in the Gastrointestinal Microbiome Under Different Temperatures

**FIGURE 2:**
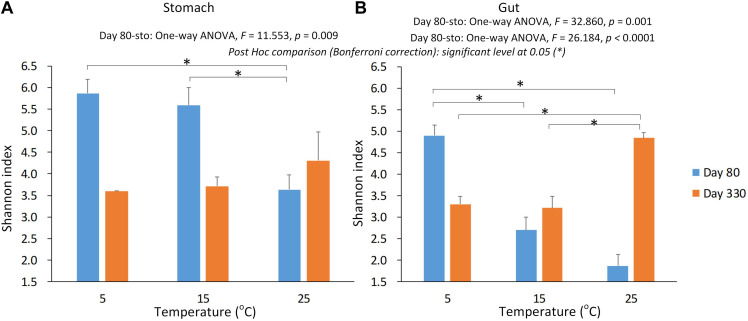
The alpha diversity of the gastrointestinal microbiome under the environmental temperatures. **(A)** The Shannon in the stomach samples. **(B)** The Shannon in the gut samples. The one-way ANOVA test was used to test the significant differences among the temperature groups within the same sample type at each sampling time. If significant, the *post hoc* test [by Bonferroni correction: significant level at 0.05 (^∗^)] was used to pairwise comparisons among the temperature groups within the same sample type. Due to only one stomach sample at 5-degree group was successfully gained the 16s data on day 330, we didn’t include this sample in the statistical analysis.

We used Shannon index to evaluate the alpha diversity of the gastrointestinal microbiome (Figure 2). We found the significant Interaction between sampling time and temperature, or type (stomach and gut) on Shannon index in the gastrointestinal microbiome of the giant salamanders (General linear model, *p* < 0.0001, Table 1). The results of one-way ANOVA test showed the significant different in Shannon index among temperature groups within the same sample type at each sample time, except for the stomach samples on day 330 (Figure 2). Because only one sample in the 5-degree group was successfully gained the 16S data. Thus, we didn’t make the one-way ANOVA test, and compared the mean value between 15-degree and 25-degree stomach groups on day 330. In the gut microbiome, the diversity in the 5-degree group was the highest on day 80, and the diversity in the 25-degree group was highest on Day 330. The trends of alpha diversity in the stomach are similar from 5-degree to 15-degree but opposite from 15-degree to 25-degree for the two sampling time points (Figure 2A). Here, we had to be cautious that there was only one sample in the 5-degree group. In the gut microbiome, the diversity under temperature 5-degree was the highest on day 80, but it decreased from 5-degree to 15-degree and then remained almost stable to 25-degree. Contrarily, on day 330, the diversity of the 25-degree group was the highest, but it presented a continuously increasing trend. Thus, the trends observed in the gut were opposite from 5-degree to 15-degree but similar from 15-degree to 25-degree for the two growth stages (Figure 2B).

**TABLE 1:** The effect on the Shannon index of the gastrointestinal microbiome by sample time (day 80 and day 330), type (stomach and gut), temperature, and their interactions.

Source	Type III sum of squares	df	Mean square	*F*	Sig.
Corrected Model	48.963^a^	10	4.896	8.132	<0.0001
	561.861	1	561.861	933.172	<0.0001
Sampling time * Temperature	13.019	2	6.509	10.811	<0.0001
Sampling time * Type	10.541	1	10.541	17.508	<0.0001
Temperature * Type	2.349	2	1.174	1.950	0.157
Sampling time * Temperature * Type	0.004	1	0.004	0.007	0.933
Error	21.676	36	0.602		
Total	814.878	47			
Corrected Total	70.639	46			

*^*a*^R Squared = 0.693 (Adjusted R Squared = 0.608). Sig., significant.*

### Effect on the Similarity in the Gastrointestinal Microbiome Community Under Different Temperatures

**FIGURE 3:**
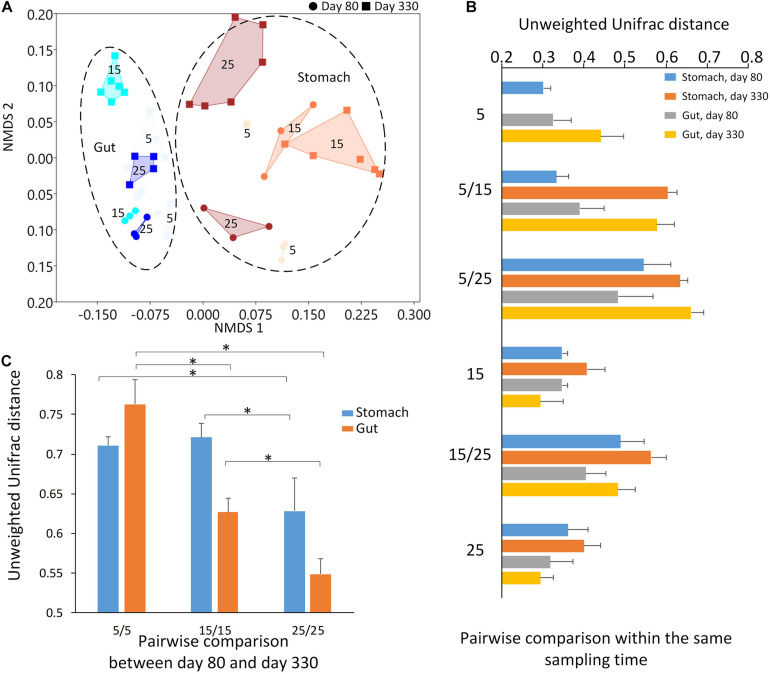
The results of beta diversity among the groups. **(A)** The NMDS (non-metric multidimensional scaling) cluster analysis using Bray-Curtis distance among all samples (each spot represented one sample). The numbers in the figure represented the degree of the temperature. The closure for each group was generated by Convex Hull (Barber et al., 1996). **(B)** The pairwise comparisons among the groups in the stomach and gut microbiome under different temperatures within each sampling time. **(C)** The pairwise comparisons between the first and second sampling time in the stomach and gut microbiome under the same temperature. The one-way ANOVA test was used to test the significant differences in the pairwise comparison distance within the same sample type. If significant, the *post hoc* test [by Bonferroni correction: significant level at 0.05 (*)] was used to pairwise comparisons.

The Adonis method and PCoA cluster using unweighted Unifrac distance showed a significant effect on the microbiome community by the sampling time (day 80 and day 330), sample type, (Type: two groups, stomach, and gut, Figure 3), and temperature (three groups: 5-degree, 15-degree, and 25-degree (Table 2, permutation = 999, *p* = 0.001), and their interaction (Table 2, permutation = 999, *p* = 0.001). For example, 10% of variation (*R*^2^ = 0.101) in microbiome community could be explained by temperature alone. In each type of the microbiome, both the sampling time and temperature had a significant effect on the microbiome community (Table 2, permutation = 999, *p* = 0.001), and the variation explained by temperature was about 16% and 20%, respectively. The variation explained by temperature was about 39% and 33%, respectively. Furthermore, within the stomach sample on first sample time, the effect of temperature explained 45.1% of the variation. These findings indicated that the temperature had a potential effect on the microbiome community besides sampling time and type.

**TABLE 2:** The Adonis analysis using unweighted Unifrac distance among the groups.

Categories	*F*	*R2*	*p*
**(1) All samples**			
Type (Stomach vs. Gut)	11.523	0.204	0.001
Sampling time (Day 80 vs. Day 330)	13.398	0.229	0.001
Temperature (5, 15, and 25 degrees)	5.071	0.101	0.001
Sampling time * Temperature	6.909	0.457	0.001
Sampling time * Type	13.603	0.486	0.001
Temperature * Type	5.577	0.405	0.001
Sampling time * Temperature * Type	10.784	0.772	0.001
**(2) Stomach**			
Sampling time (Day 80 vs. Day 330)	12.498	0.385	0.001
Temperature (5, 15, and 25 degrees)	3.765	0.158	0.010
**(3) Gut**			
Sampling time (Day 80 vs. Day 330)	11.270	0.329	0.001
Temperature (5, 15, and 25 degrees)	5.574	0.195	0.001

*Adonis: Partitions a distance matrix among sources of variation in order to describe the strength and significance that a categorical or continuous variable has in determining variation of distances (http://qiime.org/scripts/compare_categories.html). Here, the variables include sampling time, type, and temperature. (1) *All samples*: All 47 samples were used in the analysis. (2) *Stomach*: Only the samples from the stomach were used in analysis. (3) *Gut*: Only the samples from the gut were used in analysis.*

The largest unweighted Unifrac distances were found in the groups between 5-degree and 25-degree groups (Figure 3B), indicating the increasing dissimilarity in the microbiome community along with the increasing temperatures. After comparing the distance within the same type of the microbiome between the first and second sampling time under the same temperature, we found the distance decreased along with the increasing temperature, indicating the relative stability in the microbiome under the high temperature. The one-way ANOVA test showed the significant differences in the pairwise comparison distance within the same sample type (Table 2; Stomach: *F* = 40.450, *p* < 0.0001; Gut: *F* = 353.522, *p* < 0.0001). For example, the dissimilarity in the stomach and gut samples between the first and second sampling time under temperature 25 was the significant lower than that of temperature 5 or temperature 15 (Figure 3C and Table 2).

### Differences in Giant Salamander Growth Under Different Temperatures

Thermal acclimation influenced the larvae body weight and length significantly, and larvae acclimated at higher temperatures tended to have higher growth rate (*F*_2,191_ = 41.142, *p* < 0.001, two-way ANOVA; Supplementary Figures 6A–C). Further simple effect analyses indicated that significant weight and length difference between the three groups was only observed on Day_330, but not on Day_19 and Day_80 (at the threshold of *p* < 0.05; Supplementary Figures 6B,C). On Day-330, the body weight of 25-degree and 15-degree group was three and two times larger, respectively, than that of 5-degree group. The difference in body length was relatively smaller, and the 25-degree and 15-degree larvae was 1.5 and 1.3 times longer than the 5-degree individuals. There was a significant interactive effect of temperature and length on the larvae weight (*F*_2,191_ = 62.79, *p* < 0.001, ANOCOVA with length as a covariate; Supplementary Figure 6D), indicating different weight-length relationships between groups. The salamanders in the 25-degree group tended to have a larger slope in the weight-length relationship than the rest two groups (at the threshold of *p* < 0.05, LSD *post hoc* test; Supplementary Figure 6E), implying fatter body type in 25-degree individuals. In consistent with the results of growth rate, we observed a significant difference in food intake (mixed model for repeated measures, *F*_2,53_ = 39.242, *p* < 0.001) and intake rate (intake amount/total feeding food; *F*_2,53_ = 13.441, *p* < 0.001) among groups (Supplementary Figures 6F,G). And the salamanders in the 25-degree group showed the highest food intake and intake rates (pairwise LSD test, *p* < 0.05).

## Discussion

Previous studies found a decrease in alpha diversity in several animal gut microbiomes with increasing temperatures [e.g., terrestrial adult salamander (Fontaine et al., 2018), lizards (Bestion et al., 2017), hens (Zhu et al., 2019), and mice (Chevalier et al., 2015)]. However, some studies have not found significant differences in alpha diversity with increasing temperatures [e.g., the gut microbiome of tadpoles (Kohl and Yahn, 2016) and the rumen microbiome of cows (Tajima et al., 2007)]. In this study, on day 80, the alpha diversity in both the stomach and gut microbiome decreased with increasing temperatures. On day 330, the pattern was, to some extent, the opposite. Moreover, one possibility for the changes in the stomach and gut microbiome between the two sampling times is not just growth but also a change in diet (from eating worm to fish). Diet is one of the important environmental factors influencing the gut microbiome community (Backhed et al., 2005; Ley et al., 2008a,b; Xu and Knight, 2015). Thus, here, we focused on the changes in the microbiome within each sampling time.

The environmental temperatures influenced the stomach and gut microbiome communities across the two sampling times. At each sampling time, the environmental temperature explained the high percentage of variation in both the stomach and gut microbiomes among the groups. Most dissimilarities in the microbiome community were found between the 5-degree and 25-degree groups. Interestingly, the microbiome communities (either stomach and gut) displayed the highest similarity in the 25-degree group between the two sampling times when compared to those in the 5-degree and 15-degree groups. The 5-degree group had the highest dissimilarity in the microbiome community among the three groups. This finding indicates that high-temperature pressure might lead to relative stability in the microbiome during growth. However, the individuals were not the same during these two sampling times due to the invasive sampling methods for collecting stomach and gut contents. The environmental temperature disrupts the animal’s microbiome community (Chevalier et al., 2015; Bestion et al., 2017; Fontaine et al., 2018; Zhu et al., 2019). However, as a long-term effect, the high environmental temperature may act as a main selective pressure to maintain the relative stability of the microbiome along with the host development.

High environmental temperatures might influence the animal’s growth through their gastrointestinal microbiome at the long-term level. There is some consensus regarding the changes in several animal gut microbiomes with increasing temperatures: the relative abundance of Firmicutes increases, and the relative abundance of Proteobacteria decreased (Reviews in Juan and Andrew (2020)). Here, on day 330, we found the relative abundance of Firmicutes significantly increased with increasing temperature, and the ratio of Firmicutes to Bacteroidetes also increased with increasing temperatures, especially in the gut microbiome. In these ten common genera between the stomach and gut microbiome at this stage, most of them came from Firmicutes, such as *Clostridium sensu stricto 1*, *Dielma*, *Hydrogenoanaerobacterium*, *Intestinibacter*, *Intestinimonas*, *Oscillibacter*, *Peptococcus*, and *Ruminococcaceae UCG-014*. In the human and mice gut microbiome, Firmicutes are more effective as an energy source than *Bacteroidete*s, thus promoting absorption of calories and nutrient transportation, and subsequent weight gain (Turnbaugh et al., 2006; Krajmalnik-Brown et al., 2012; Dugas et al., 2016). In this temperature study, the larvae of the salamander were initially overfed with commercial red worms daily. After 254 days of acclimation, the larvae were overfed with fresh fish every 2 days. We have compared the composition of redworm and fish; the latter is much richer in protein and lipid. Therefore, given that Firmicutes are highly correlated with protein and fat digestibility, we speculated that the significant increase in the relative abundance of Firmicutes in the gastrointestinal microbiome with increasing temperatures on day 330 might be associated with the high food intake and intake rate, which resulted in the significantly increased body weight and length of the giant salamanders. Here, we have to mentioned that higher temperatures led to increased host metabolism and food consumption, which might result in a higher level of nutrients for the gut microbiome and the changes in the gut microbiome community. Thus, our study might still be an indirect consequences of the temperature increase on the gut microbiome, but it did make the change in microbiota a secondary effect, rather than a direct response.

It should be pointed out that the acclimation temperature grads (5, 15, and 25°C) used in this study was based on the hatchery temperature (15.18 ± 1.14°C) of the artificial populations in Hongya County in Sichuan Province, China. These larvae prefer temperature of 10–15°C (Unpublished data). In fact, the optimum thermal window of captive *A. davidianus* larvae may vary with the clade (Yan et al., 2018) and breeding conditions. For example, previous studies have reported that 20–24°C are suitable for the growth of *A. davidianus* individuals bred at 20°C (Hu et al., 2016, 2019a,b). There were some limitation in this study. Firsts, we didn’t have the samples before the temperature treatment, and we didn’t know the baseline in the microbiome community for these groups. Second, we couldn’t follow individuals across time due to the invasive genetic methods used in this study. Thus, the variation among the individuals would also lead to the difference in the microbiome composition. The best way in this kind of research would use the non-invasive genetic method to track the microbiome composition of each individual across time under the different temperature treatment.

## Conclusion

Here, based on the analysis of the gastrointestinal microbiome in the laboratory larva of giant salamanders, we revealed that the environmental temperature might have an indirect effect on the microbiome community. The significant increase in the relative abundance of Firmicutes in the gastrointestinal microbiome with increasing temperatures might be associated with the significantly increased host metabolism and food consumption. The high environmental temperature might act as a selective pressure to maintain the relative stability of the microbiome during the host development. However, we did not find evidence of how environmental temperature directly influences the gut microbiome.

## Materials and Methods

### Experimental Design

The Chinese giant salamander (*A. davidianus*), is an endangered species in China. It is a model species for evolutionary studies according to high starvation tolerance, their longevity, and their ability to hatch in the absence of sunshine (Geng et al., 2017). Larvae of the giant salamander (104 days after hatching, 3.26 ± 0.05 g) were bought from an artificial farm located in Hongya County, Sichuan province in China on February 8, 2018. On the same day, these larvae were randomly divided into three groups (30 individuals per group) and acclimated in three artificial climate boxes at 5 ± 0.5°C, 15 ± 0.5°C (empirical optimum in the farm), and 25 ± 0.5°C (water temperature; 12L: 12D). We also manipulate the daily condition with the 12-hourly light and 12-hourly dark per day. We used these temperatures to represent cold, optimal, and heated living conditions and these salamander typically experience temperatures of 8–25°C, with 15–21°C as optimal (Hill et al., 1975; Hutchison and Hill, 1976). The breeding center is normally set up at 15°C for their living water temperature. If the water temperature is below 8°C, the giant salamander will lose appetite, which leads to a decrease in the growth rate. If the water temperature is below 3°C or over 28°C, the giant salamander will stop eating and go into a hibernation status (Mu et al., 2011). These larvae were initially overfed with commercial red worms (*Limnodrilus*) daily. After 254 days of acclimation, the larvae were overfed with fresh fish every 2 days. Food intake was measured by the difference between the initial and remnant food mass. Water was replaced before each feeding.

The experiment was designed to study the chronic influences of temperature on the physiology and metabolism of the giant Chinese salamander, and the reorganization of the microorganisms was an important aspect. The reason why we called it “acclimation” was because some physiological tests were conducted during or at the end of the thermal exposure. These included comparisons of the preference temperatures and thermal tolerant windows between larvae acclimated at different temperatures. Since the larvae grew up, the redworm could no longer provide sufficient nutrients to the individuals acclimated at higher temperatures (delayed somatic growth), but fish did. This is the experience of the farmers and our observations. On the farm, the hatched larvae were fed with red worm for the first several months, and then this diet was replaced by the pork liver or fish. We have compared the composition of redworm and fish; the latter is much richer in protein and lipid. The salamanders acclimated at the same temperature were kept in three independent containers/tanks, and each container housed ten individuals. On first sample timing, five individuals were collected randomly from each container, and thus 15 individuals were collected for each thermal group. On second sample timing, two individuals were collected randomly from each container, and thus six individuals were collected for each thermal group.

### Sample Collection

The stomach and gut contents per individual were collected after 80 and 330 days of acclimation. For stomach and gut microbial sampling, each giant salamander was euthanized and dissected to collect the gut and stomach contents in a 2 mL aseptic centrifuge tube. Due to the lack of sufficient gastrointestinal content in a single individual on day 80, we collected nine pooled samples for the stomach (three for each temperature) and nine pooled samples for the gut (three for each temperature). Each pooled sample was collected from five individuals from the same container. Before sample collection, larvae were randomly selected from each tank and fasted for 3 days. The entire content of each stomach was collected after removing the food residue, and the entire intestine content (from the anterior intestine to the cloaca) was collected as gut sample. Each sample was mixed by several rounds of vortex and transient centrifugation. On day 330, we did not use a pooling strategy and collected stomach and gut contents from each individual. We collected the stomach and gut contents from six individuals for each temperature (However, in the sequencing, two failed in 15-degree gut group, two failed in 25-degree gut group, five failed in 5-degree stomach group). On the second sampling time, the larvae were much larger in size, but the stomach and gut content collected did not exceed 2 ml for each individual. All samples were stored at −80°C until DNA extraction. On day 80, we obtained nine pooled stomach and nine pooled gut samples from 45 individuals, and each temperature group had three pooled samples within the stomach and the gut microbiome. On day 330, we successfully obtained 29 samples, and each temperature group had 1–6 samples.

### DNA Extraction and 16S rRNA Sequencing

Each sample was thawed on ice, and microbial genomic DNA was extracted using a QIAamp Fast DNA Stool Mini Kit (QIAGEN, Germany), according to the manufacturer’s protocol. The negative controls (blank control, no adding the gut or stomach sample) were used during the DNA extraction. The integrity of the DNA was visually assessed using 1.0% agarose gel electrophoresis and quantified using a Qubit and NanoDrop. The 16S rRNA gene V4 region was amplified from the extracted DNA using the universal primers 515F (GTGCCAGCMGCCGCGGTAA) and 806R (GGACTACHVGGGTWTCTAAT). The PCR was performed in triplicate using a 25 μL reaction containing ∼40 ng of DNA template, 2.5 μL of 10x TransStart Taq buffer, 1 μL of each forward and reverse primer, 2 μL of dNTPs (2.5 mM), 0.25 μL of TransStart Taq DNA Polymerase, and 16.25 μL of ddH_2_O. The polymerase chain reaction thermocycling conditions were: 95°C for 5 min, 35 cycles of 95°C for 30 s, 55°C for 30 s, and 72°C for 45 s, with a final extension step at 72°C for 10 min. All the PCR products from the blank controls (blank extraction control and blank PCR control) were blank in the agarose gel. The products were purified with a DNA Purification Kit (TIANGEN, Beijing), and barcoded V4 amplicons were sequenced using the Illumina HiSeq platform (HiSeq2500 PE250).

### Analysis of 16S rRNA Raw Data

#### Raw Read Trimming

Here, we used QIIME 1.9 to trim the raw reads and obtain clean sequences (Caporaso et al., 2010). In the trimming analysis, *Usearch* was used for chimerism check in order to remove low-quality sequences, *flash* was used for splicing, and *trimmomatic* was used for quality control with default parameters (e.g., Window Size: 20 base pair; Minimum Read Length: 50 base pair) (Edgar, 2010). Operational taxonomic units (OTUs) were defined as sharing >97% sequence identity, and representative sequences were classified against the SILVA132 database (Quast et al., 2012). Then, we obtained OTU tables containing taxon information (e.g., Phylum, Class, Order, Family, and Genus). We chose to rarefy our sequencing depth at 28202 (according to the lowest number of sequences of one sample in this study) to equalize the sampling depth across all samples. The taxon summary of these sequences mostly were assigned to Bacteria domain, and only about 0.5% were unclassified.

#### Alpha Diversity Analysis

The alpha diversity (e.g., Shannon index) was calculated in QIIME 1.9 (Caporaso et al., 2010). The general linear was used to evaluate the interaction between day and temperature, or with sample type. We use one-way ANOVA test to test the significant differences among the temperature groups within the same sample type at each sampling time. If significant, the *post hoc* test (by Bonferroni correction) was used to pairwise comparisons among the temperature groups within the same sample type. In addition, due to only one stomach sample at 5-degree group was successfully gained the 16s data on day 330, we didn’t include this sample in the statistical analysis. Thus, non-parametric analysis was used to compare the mean value of Shannon index between the 15-degree and 25-degree groups within the stomach samples. All statistical analysis were conducted in SPSS Statistics 20.0 (Spss, 2011).

#### Estimating the Dynamics of the Microbiome at Different Temperatures

We used linear discriminant analysis effect size (LEfSe) (Segata et al., 2011) to determine the stomach and gut microbial taxa (genus level) with significantly differentiating relative abundance among groups (under different temperatures) within each sampling time. Thus, within each sample time, we used this genera information (including the mean relative abundance) to find four types of trends in each type of the microbiome with increasing temperatures: increasing (from 5 to 25 degree), decreasing (from 5 to 25 degree), convex (increasing from 5 to 15 degree, and then decreasing in 25-degree), and concave patterns (decreasing from 5 to 15 degree, and then increasing in 25-degree).

#### Microbial Beta Diversity Analysis

Muscle (Edgar, 2004) was used to make the alignment of the represented sequences of each OTU, and FastTree (Price et al., 2010) was used to construct the phylogenetic tree. We applied Adonis method in QIIME 1.9 (Caporaso et al., 2010) based on the unweighted Unifrac distance using OTU tables to compute an *R*^2^ value (effect size), which showed the percentage of variation explained by factors (e.g., sampling time, type), and their interaction. The Bray-Curtis distance for bacterial species abundance was used to generate NDMS (non-metric multidimensional scaling) in PAST3 (Hammer et al., 2001). At each sampling time, pairwise comparisons based on unweighted Unifrac distances between different temperature groups in the stomach and the gut microbiome were generated with QIIME 1.9 (Caporaso et al., 2010). Furthermore, we made comparisons based on unweighted Unifrac distances in the same temperature group between the first and day 330s within the stomach and the gut microbiome. The one-way ANOVA test was used to test the significant differences in the pairwise comparison distance within the same sample type. If significant, the *post hoc* test (by Bonferroni correction) was used to pairwise comparisons.

### Growth Analysis of the Giant Salamander

Bodyweight and length were measured every week. The food intake rate was calculated using the following formula: food intaking/total feeding food. Repeated measures models (general linear model or mixed model) could not be conducted for larvae body traits, as larvae from the same tank were not marked, and thus the data could not be distinguished between individuals. Here, in order to test the differences in the growth characters among the groups (under the different living temperatures), we tried to analyze these metrics. Pairwise comparisons of the body traits at each sampling time point were followed by a one-way ANOVA LST test. The difference in weight to length ratio was also analyzed between groups. All tests were conducted in SPSS Statistics 20.0 (Spss, 2011).

## Data Availability Statement

The datasets presented in this study can be found in online repositories. The names of the repository/repositories and accession number(s) can be found below: NCBI BioProject, accession no: PRJNA659382.

## Ethics Statement

The animal study was reviewed and approved by the Animal Ethical and Welfare Committee of Chengdu Institute of Biology, Chinese Academy of Sciences.

## Author Contributions

LZ and JJ conceived the project. WZ, CZ, LX, and TZ collected the samples. WZ, CZ, and LX performed the experiments. LZ, HC, and WZ analyzed the data. LZ, QC, and JJ wrote the manuscript. All authors provided final approval for publication.

## Conflict of Interest

HC was employed by company Mingke Biotechnology Co., Ltd., Hangzhou, China. The remaining authors declare that the research was conducted in the absence of any commercial or financial relationships that could be construed as a potential conflict of interest.
