# Supplementary material for: Environmental Temperatures Affect the Gastrointestinal Microbes of the Chinese Giant Salamander
Source: Front Microbiol. 2021 Mar 19;12:543767. doi: 10.3389/fmicb.2021.543767 (PMC8017128; doi:10.3389/fmicb.2021.543767)
Supplement: Supplementary file 1 [file Data_Sheet_1.docx]

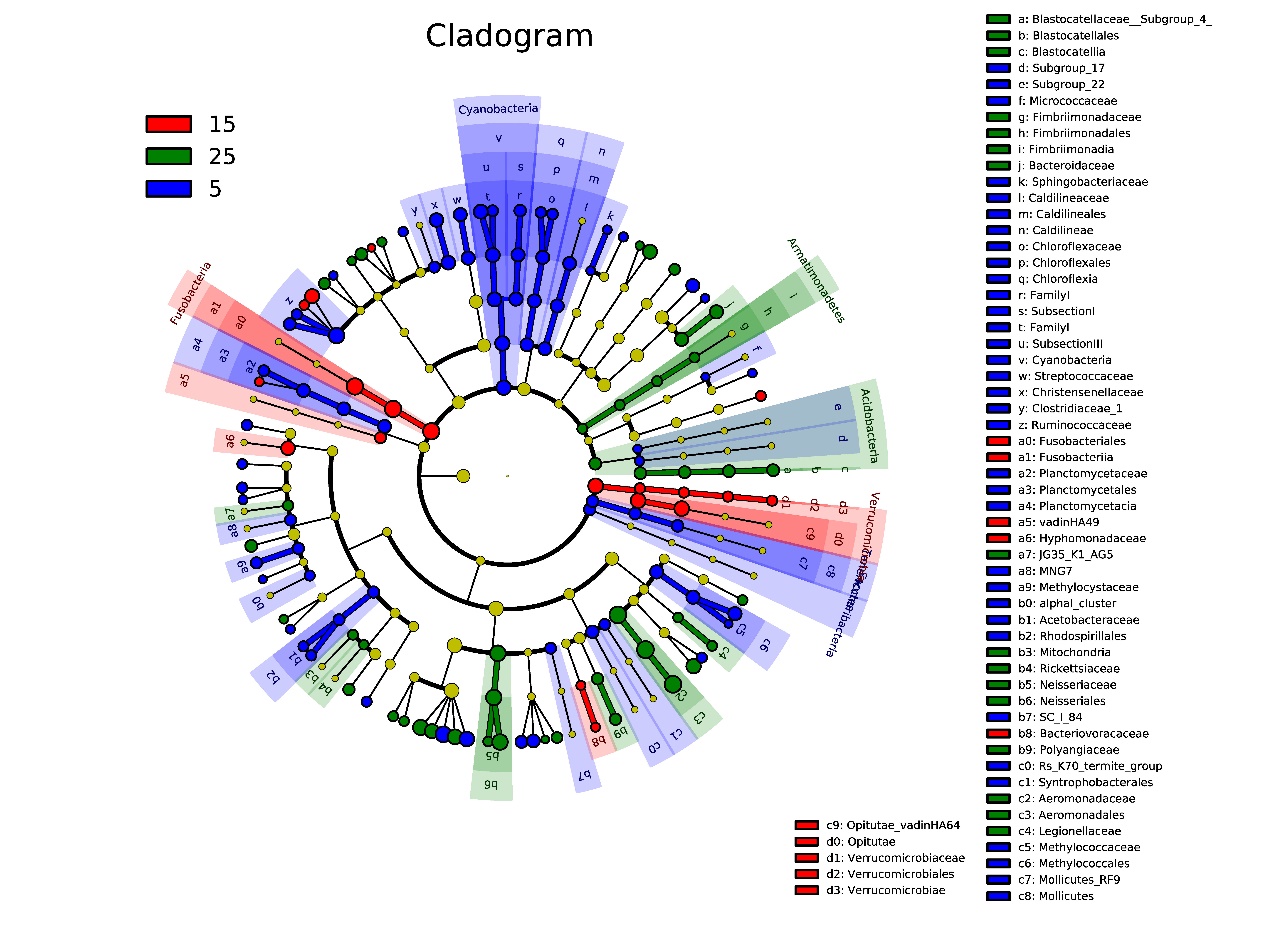


**Figure S1** The LEfSe (Linear discriminant analysis Effect Size) analysis in the stomach microbiome community among groups at Days_80. Days_80, 80 days after acclimation. 5, 5 ^o^C, 15, 15 ^o^C. 25, 25 ^o^C.


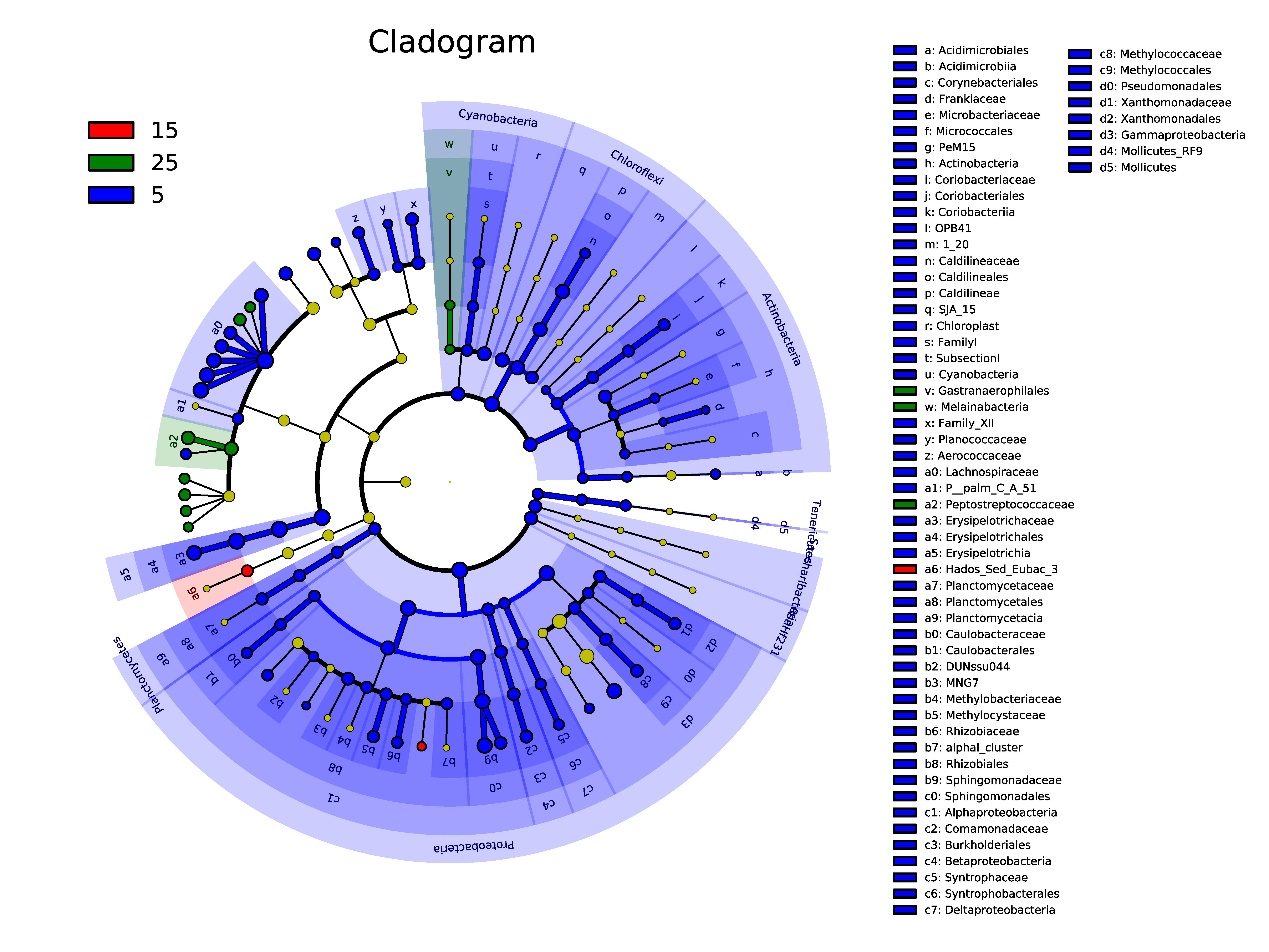


**Figure S2** The LEfSe (Linear discriminant analysis Effect Size) analysis in the gut microbiome community among groups at Days_80. Days_80, 80 days after acclimation. 5, 5 ^o^C, 15, 15 ^o^C. 25, 25 ^o^C.


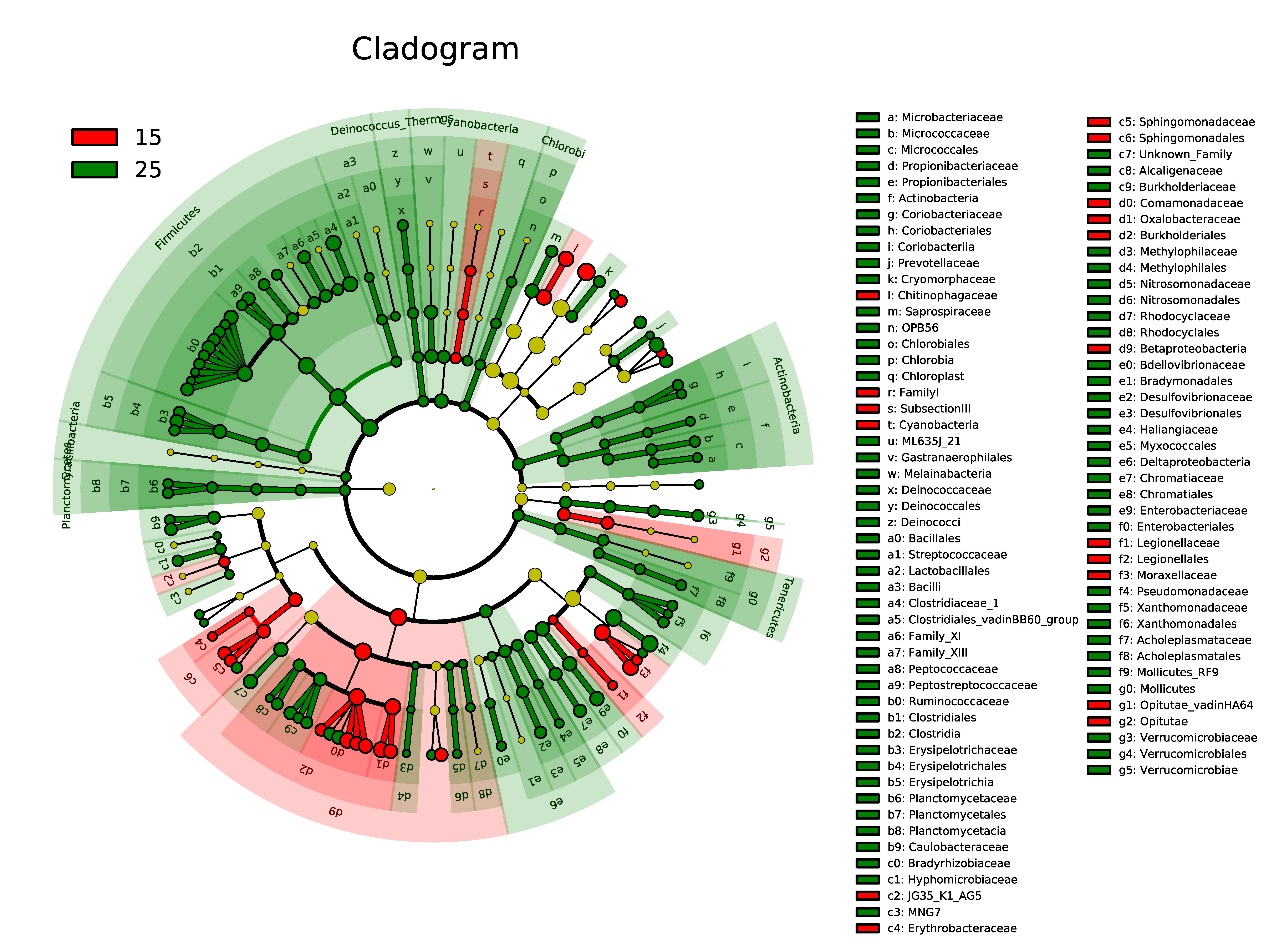


**Figure S3** The LEfSe (Linear discriminant analysis Effect Size) analysis in the stomach microbiome community among groups at Days_330. Days_330, 330 days after acclimation. 5, 5 ^o^C, 15, 15 ^o^C. 25, 25 ^o^C.


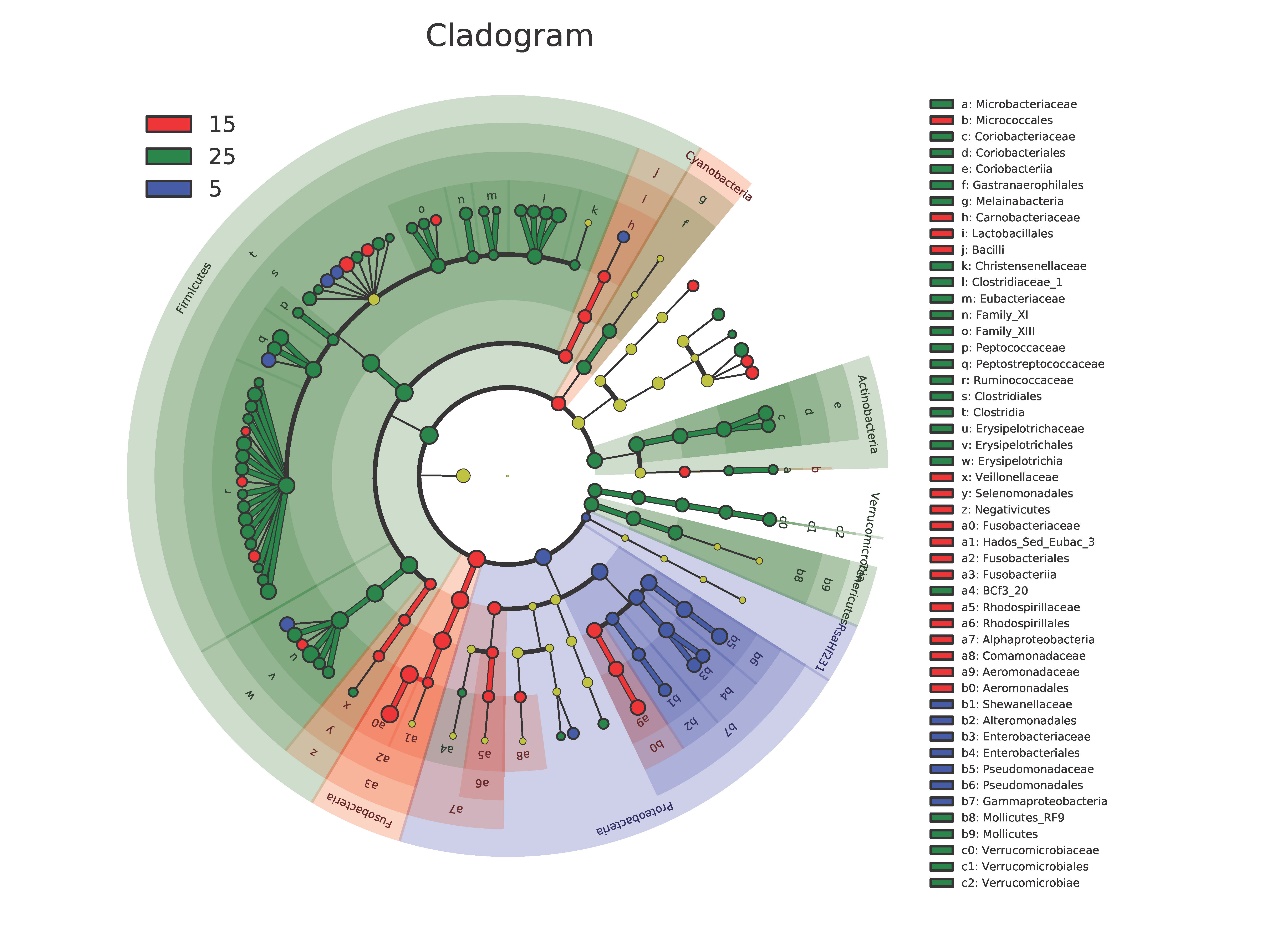


**Figure S4** The LEfSe (Linear discriminant analysis Effect Size) analysis in the gut microbiome community among groups at Days_330. Days_330, 330 days after acclimation. 5, 5 ^o^C, 15, 15 ^o^C. 25, 25 ^o^C.


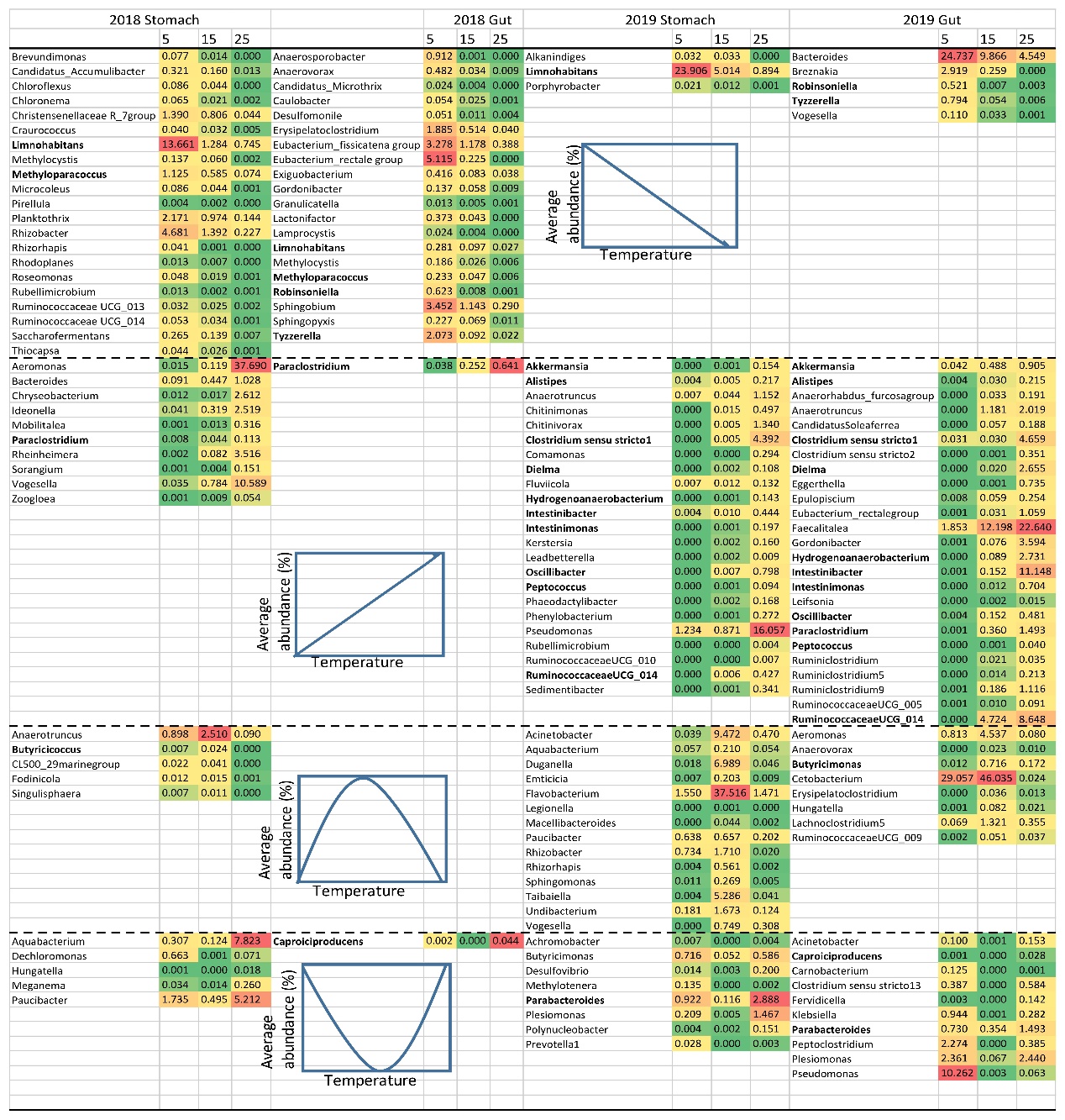


D

C

B

A

**Figure S5** Changes in the abundance of the symbiotic microbiome (genus level) among the groups based on the LEfSe analysis. Days_80, 80 days after acclimation. Days_330, 330 days after acclimation. 5, temperature 5 ^o^C. 15, temperature 15 ^o^C. 25, temperature 25 ^o^C.

A, the increasing pattern from 5-degree to 25-degree. B, the decreasing pattern from 5-degree to 25-degree. C, convex pattern (increasing from 5-degree to 15-degree, and then decreasing in 25-degree). D, concave patterns (decreasing from 5-degree to 15-degree, and then increasing in 25-degree). The colors from green to red (heatmap) represented the increasing in the relative abundance.


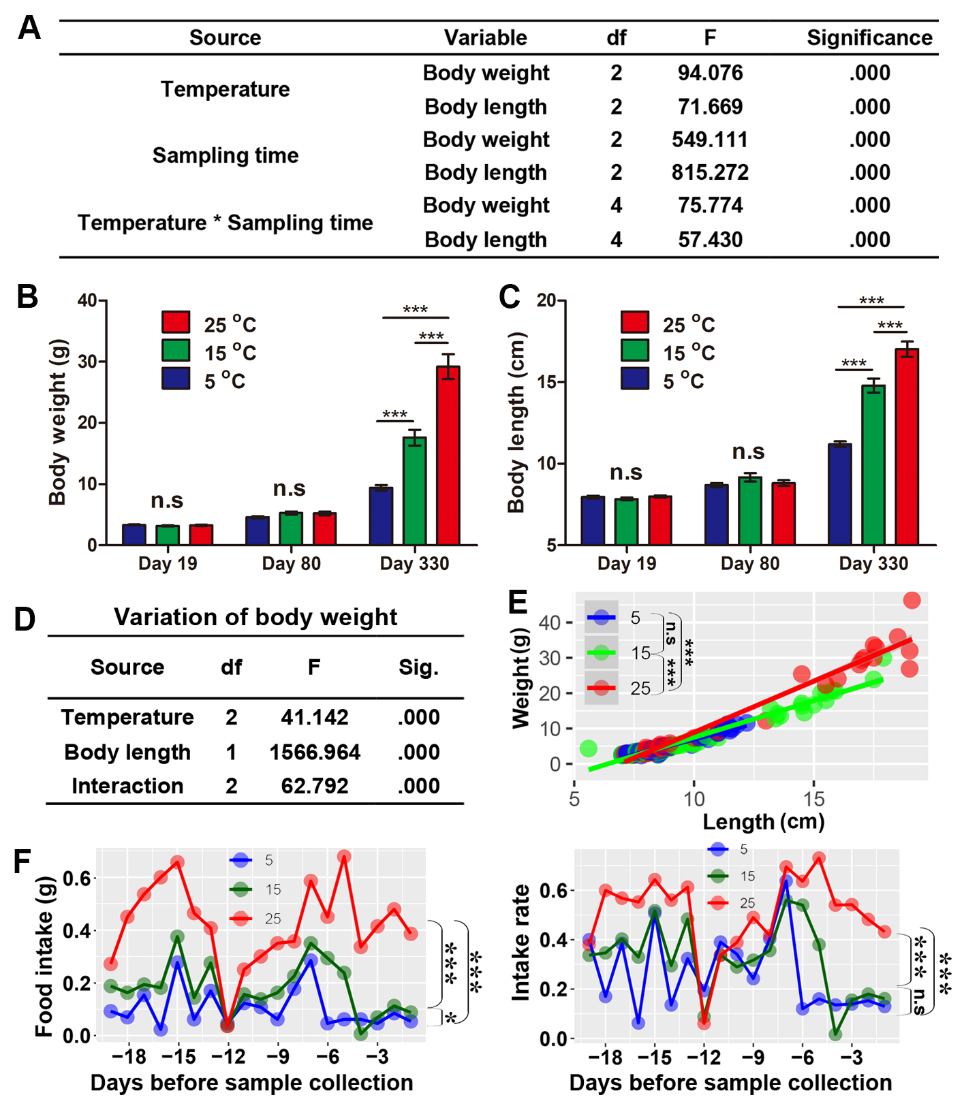


**Figure S6 Growth of the giant salamanders under different temperatures.** (A-C) Variation of body weight and length between thermal groups. Data was analyzed by two-way ANOVA and LSD post-hoc test, with temperature and sampling timepoints as fixed factors. ***, *p* < 0.001. (D-E) Influence of temperature on the relationship between body weight and body length of giant salamanders. Data was analyzed with a covariance analysis, with the weight as dependent variable, temperature as fixed factor, and the length as a covariant. Note the small p value of the interactive effect of temperature and body length on body weight, which indicates different length-weight slopes between thermal groups. ***, p < 0.001. (F-G) The amount of food intake (F, per individual) and food intake rate (G) at the second sampling time. Data was analyzed with repeated measures mixed model. *, *p* < 0.05; ***, *p* < 0.001.
